# Integration of Semi-Circular Canal and Otolith Cues for Direction Discrimination during Eccentric Rotations

**DOI:** 10.1371/journal.pone.0136925

**Published:** 2015-08-31

**Authors:** Florian Soyka, Heinrich H. Bülthoff, Michael Barnett-Cowan

**Affiliations:** 1 Max Planck Institute for Biological Cybernetics, Department: Human Perception, Cognition and Action, Tübingen, Germany; 2 Department of Brain and Cognitive Engineering, Korea University, Anamdong, Seongbuk-gu, Seoul, Korea; 3 Department of Kinesiology, University of Waterloo, Waterloo, Ontario, Canada; VU University Amsterdam, NETHERLANDS

## Abstract

Humans are capable of moving about the world in complex ways. Every time we move, our self-motion must be detected and interpreted by the central nervous system in order to make appropriate sequential movements and informed decisions. The vestibular labyrinth consists of two unique sensory organs the semi-circular canals and the otoliths that are specialized to detect rotation and translation of the head, respectively. While thresholds for pure rotational and translational self-motion are well understood surprisingly little research has investigated the relative role of each organ on thresholds for more complex motion. Eccentric (off-center) rotations during which the participant faces away from the center of rotation stimulate both organs and are thus well suited for investigating integration of rotational and translational sensory information. Ten participants completed a psychophysical direction discrimination task for pure head-centered rotations, translations and eccentric rotations with 5 different radii. Discrimination thresholds for eccentric rotations reduced with increasing radii, indicating that additional tangential accelerations (which increase with radius length) increased sensitivity. Two competing models were used to predict the eccentric thresholds based on the pure rotation and translation thresholds: one assuming that information from the two organs is integrated in an optimal fashion and another assuming that motion discrimination is solved solely by relying on the sensor which is most strongly stimulated. Our findings clearly show that information from the two organs is integrated. However the measured thresholds for 3 of the 5 eccentric rotations are even more sensitive than predictions from the optimal integration model suggesting additional non-vestibular sources of information may be involved.

## Introduction

Reliable and robust self-motion perception is essential for everyday life since we are constantly moving about in the world. Understanding exactly how the central nervous system creates robust percepts from available sensory information is crucial in order to be able to assess and treat neural disorders as well as to design motion simulators or vestibular prostheses that are able to accurately induce desired self-motion percepts. One of the fundamental methodologies for investigating self-motion perception in humans is the measurement of perceptual thresholds. Thresholds have been intensively studied for translational motions [1–4] as well as rotational motions [5–8] and a good understanding of how thresholds vary with the presented motion profile has been achieved. However, thresholds for more complex motions that include both translational and rotational components have received less attention [9]. The goal of the present work is to systematically investigate how thresholds change as the ratio of translational/rotational cue intensity varies. Specifically we try to distinguish between two alternative scenarios: either canal and otolith cues are integrated in order to form a percept or the stronger cue dominates perception.

**Fig 1:**
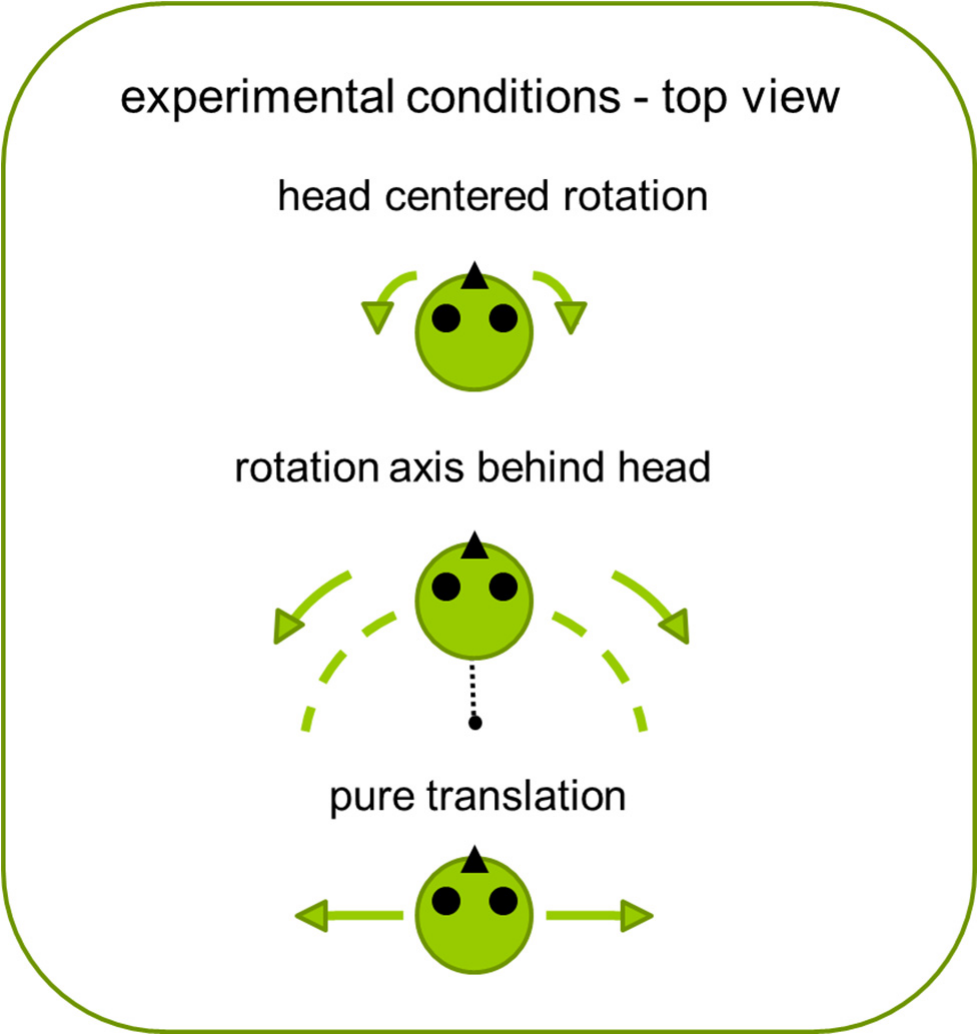
Conditions. Description of the experimental conditions as they were presented to the participants.

In this study, we used a direction discrimination task in which participants had to judge whether a motion was leftward or rightward and thresholds were measured for translational motions, head-centered rotations and eccentric rotations (Fig 1). Eccentric rotations (also known as off-center rotations) are motions with a circular trajectory in which the center of rotation is located behind the participant and participants are facing away from the center of rotation. Such motions include both translational and rotational cues and are therefore well suited for studying potential cue integration, or dominance.

It has been shown that direction discrimination of passive whole-body motions in the dark is dominated by the vestibular system [4,10]. The vestibular labyrinth consists of two unique sensory organs: the semi-circular canals (SCC) and the otolith organs (OO) that are specialized to detect rotation and translation of the head, respectively. By having an eccentric (not head-centered) rotation, a tangential acceleration is introduced which is detectable by the otolith organs in addition to the rotational signal detectable by the semi-circular canals. This tangential acceleration depends on the direction of the eccentric rotation and therefore provides additional information that can be used to solve the direction discrimination task. Note that there is also a centrifugal acceleration present in the motion stimulus, which points away from the center of rotation. However, this centrifugal acceleration does not depend on the direction of the motion and therefore it can be neglected since it is not relevant for a direction discrimination task.

**Fig 2:**
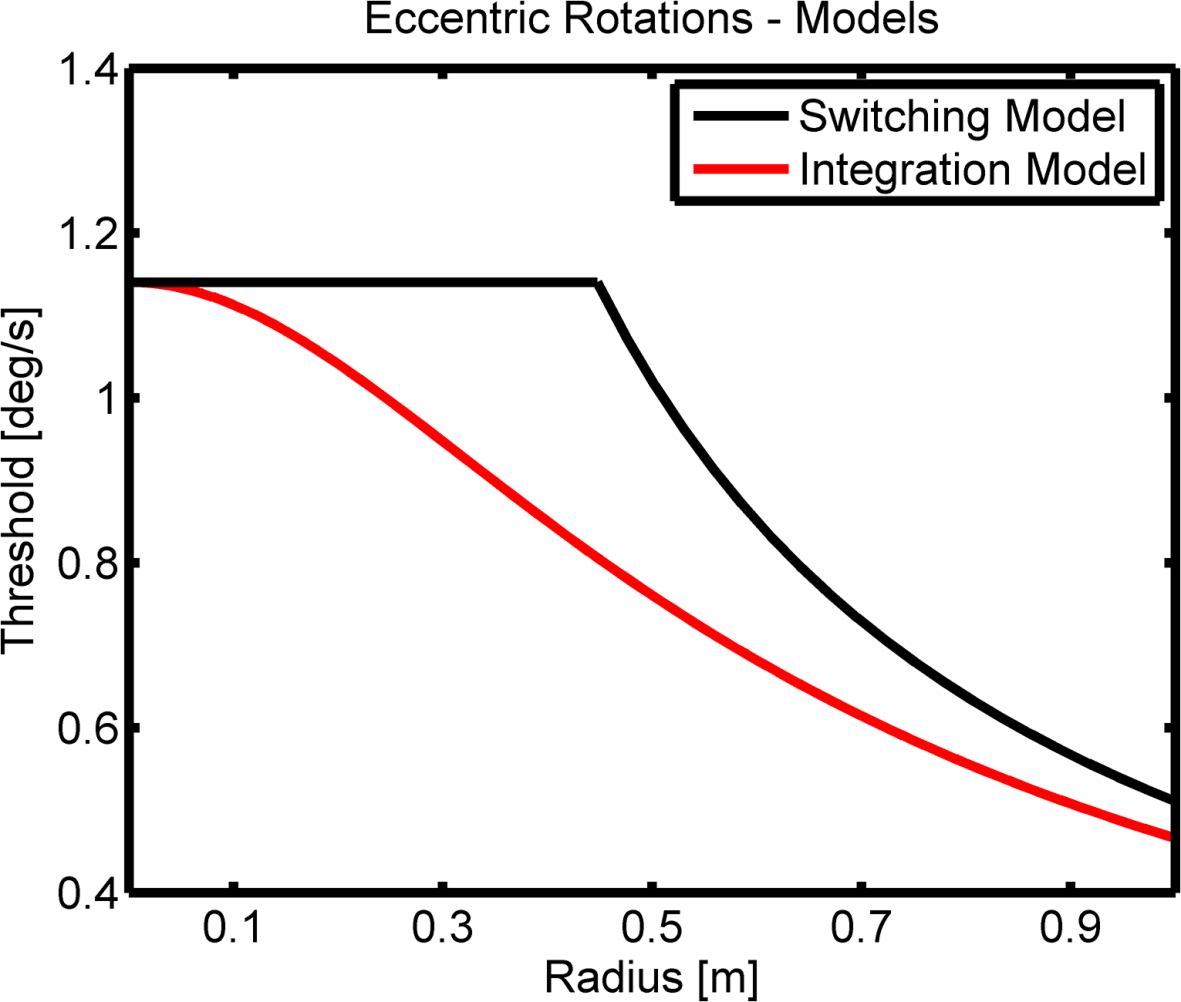
Models. Description of the two alternative models which we refer to as the switching model and the integration model. For both models less rotational velocity is needed with increasing radius for discriminating the motion direction. However for intermediate radii there are clear differences in threshold behavior between the models, where an integration model is more sensitive.

The strength of the tangential acceleration *t* for a given rotational acceleration *α* depends on the radius *r* of the rotation: *t* = *r* ⋅ *α*. Therefore, it should be possible to observe a transition from SCC dominated direction of motion discrimination to OO dominated discrimination by increasing the radius of the eccentric rotation and thereby increasing the tangential acceleration. For intermediate radii there is a regime in which SCC and OO cues are similar in perceived intensity. This range is most interesting for the purpose of the experiment, because it will help identify whether the cues from both organs are integrated or whether discrimination performance is dominated by the stronger cue. Fig 2 illustrates the two alternatives which we refer to as the switching and the integration model. With the switching model the threshold stays constant up until the radius at which the tangential acceleration becomes larger than the threshold for discriminating translational motions. After this "switching radius", the rotational threshold decreases continuously with increasing radius since less rotational acceleration is needed in order to achieve the necessary tangential acceleration for OO dominated detection. In the integration model the rotational and the translational cues are optimally combined based on each cue's relative reliability and therefore the thresholds for discriminating the direction of eccentric rotations begin to decrease already for small radii where performance for intermediate radii is better than with the switching model. For large radii the thresholds predicted by both models converge again because the translational cue is so strong that the additional rotational cue does not increase discrimination. Measuring the threshold behavior for small radii eccentric rotations will therefore allow us to discriminate between these two alternatives. Detailed model descriptions are provided in the Methods section.

To our knowledge how perceptual thresholds for direction discrimination change during eccentric rotations and whether and how cues from the SCC and the OO are integrated have not been previously investigated. Eccentric rotations have been researched in the context of centrifugation [11,12]. However, in these experiments the participant is often aligned with the direction of motion and there are periods of prolonged constant centrifugation whereas our experiment investigates more natural and transient eccentric rotations of one second duration. Vestibulo-ocular reflexes (VORs) have been studied during eccentric rotations where it has been found that both rotational and translational cues are integrated and contribute to the resulting VORs [13–15].

MacNeilage et al. [9] looked at canal-otolith interaction for detection thresholds during curved-path motion and found that translation detection thresholds become significantly worse with increasing concurrent angular motions. Note that here, the curved-path motion used in their study was not an eccentric rotation, but also stimulated SCC and OO at the same time. Furthermore the task was different: participants did not make a judgment about the whole motion percept, but were asked to only judge translational motion while there was concurrent rotational motion. The authors suggest that “conscious perception might not have independent access to separate estimates of linear and angular movement parameters during curved-path motion. Estimates of linear (and perhaps angular) components might instead rely on integrated information from canals and otoliths” [9]. Assessing whether canal and otolith cues are integrated or, alternatively, drive self-motion perception based on their relative dominance is the purpose of the present paper.

Another study looking at the ability to reproduce self-rotations found that for off-center trials (eccentric motions) the additional information from the OO seemed to interfere with the reproduction of movement duration [16]. This represents an instance in which additional information from the OO hindered rather than helped this specific task. In general, these examples demonstrate that the canal-otolith interaction is not trivial and that there might not be independent perceptual access to either the SCC or the OO signals, but rather that the resulting percept might rely on integrated information from both organs.

Integration of canal-otolith signals is also present in another crucial function of the vestibular system, the distinction between gravitational and inertial accelerations whereby ambiguous otolith signals, that alone cannot be used to distinguish tilt relative to gravity from linear translation, are integrated with semi-circular canal signals and compared to internal representations of gravity [17,18]. Consequently, models describing eye movements or perception have to take this interaction into account as well [19,20]. Both perception and action processes are determined by sensory signals from the vestibular system, however differences in the response dynamics for perception and action have been reported and might reflect additional involvement of central processing [21–26]. It is thus necessary to study how specifically information is integrated in different scenarios in order to better understand the neural mechanisms subservient of perceived self-motion. Note that both the switching and integration models assume that there is independent access to the tangential component of the gravito-inertial force vector. The implications of this assumption will be reviewed in more detail in the Discussion.

Here we measured how thresholds for eccentric rotations change with increasing radii and to distinguish between two possible scenarios: either the information from both organs is integrated (“integration model”) during direction discrimination or the decision is made based on the stronger of the two signals (“switching model”). In order to distinguish between the two scenarios we first measured thresholds for translations, for rotations and for five eccentric rotations with increasing radii in order to observe the transition between SCC dominated detection to OO dominated detection. Next, the thresholds obtained for the translation and rotation conditions are used in order to predict the eccentric rotation thresholds and these predictions are compared to the observations.

## Methods

### Participants

Ten participants (5 female) took part in the study. They were 21–29 years old and reported no vestibular problems. The participants were paid a standard fee and signed a consent form prior to the experiment. They did not receive any feedback about their performance during the study.

### Ethics Statement

The experiment was conducted in accordance with the requirements of the Helsinki Declaration and all procedures were reviewed and approved by the ethics committee of the Eberhard-Karls-Universität Tübingen.

### Motion Stimuli

Participants were tested in 7 conditions: a lateral translational motion, a head-centered yaw rotation and 5 eccentric rotations with different radii. The center of rotation of the eccentric rotations was located behind the participant and the 5 radii were: 0.1, 0.2, 0.3, 0.5 and 0.8 meters. The motion profiles had durations of 1 second (1 Hz) and were sinusoidal accelerations with varying peak amplitudes: $A\;\text{sin}\;\left(\frac{2\pi }{1s}t\right),\;0s\leq t\leq 1s$. The sinusoidal acceleration profile is also known as a *raised cosine bell* profile (referring to its shape in the velocity domain). For this specific type of stimulus peak acceleration equals peak velocity times Pi and therefore one can easily convert between acceleration and velocity units. This is useful since usually rotational thresholds are reported in terms of velocity, but due to the fact that we are interested in the relation between tangential and rotational acceleration we prefer to use acceleration units for describing rotations. Short 1Hz motions were chosen in order to avoid having the translational components interpreted as tilt [27] or having velocity storage mechanisms influence the perception of rotation [28]. Based on threshold knowledge from previous experiments [1,5] and preliminary testing data, the radii were chosen in such a way that the transition between SCC dominated detection to OO dominated detection can be readily observed.

Stimulus intensities ranged between 0.2 and 40 deg/s^2^ for the head-centered and the eccentric rotations and between 0.001 and 0.14 m/s^2^ for the lateral translations. Since the eccentric motions consist of both rotational and translational components they could also be described in terms of their tangential acceleration *t* instead of their rotational acceleration *α*. Both are related through the radius *r* of the motion: $t=r\cdot \alpha \cdot \frac{\pi }{180}$ (The factor $\frac{\pi }{180}$ converts from degrees to radians). Therefore tangential accelerations ranged between 0.0003 m/s^2^ and 0.07 m/s^2^ for r = 0.1 and 0.0028 m/s^2^ and 0.56 m/s^2^ for r = 0.8. Note that the tangential accelerations also follow the same sinusoidal profile as the rotational accelerations. We report both parametrizations (rotational and translational) for the eccentric rotations in the results in order to clarify their relationship. For the sake of simplicity we only use the rotational parameterization for the modeling. However the whole analysis could also be done using the translational parameterization resulting in the same conclusions.

In order to present motion stimuli to participants, we used the Max Planck Institute CyberMotion Simulator. Further details on its hardware and software specifications are available (Robocoaster, KUKA Roboter GmbH, Germany; [29–32]). Since the setup in this study is similar to those used in our previous works measuring perceptual self-motion thresholds, we refer the interested reader to [1,5,33] for further information.

### Experimental Procedures

A within-participants design was employed. In order to counterbalance possible learning effects, the presentation sequence of the conditions was varied between participants (sequence can be found in the supplementary material [34]). Participants were seated in a chair with a 5-point harness and wore light-proof goggles. They were instructed to keep their head still, and additionally, a neck brace was used in order to restrict head movements. Acoustic white noise was played back during the movements via headphones. Participants wore clothing with long sleeves and trousers, and a fan was directed towards the participant’s face to mask possible air movement cues during movement of the simulator. Participants were tested in two sessions of 2 hours each on two separate days. Each condition consisted of 150 direction discrimination trials and took approximately 15 minutes with a 5 minute break in between conditions in order to prevent fatigue. Participants were informed about the type of motion they would experience before each condition in order to ensure that they understood the task. They received verbal explanations about the upcoming motion and saw the pictures shown in Fig 1. They knew what kind of motion to expect in each condition and therefore there was no ambiguity in the instructions. Participants reported the direction of motion, irrespective of it consisting of translational or rotational cues.

A one-interval two-alternative forced-choice task was used to measure the psychometric function for direction discrimination. Participants initiated a trial with a button press and, after a one second pause, the movement began. They were moved either leftward or rightward, were instructed to indicate the direction of their motion as fast as possible via a button press, and were then moved back to the starting position with the same speed as the stimulus previously delivered. In total there was at least a two second period of standstill between consecutive motions (longer if participants did not immediately start the next trial).

### Psychometric Function Estimation

A Bayesian adaptive procedure, based on the method proposed by Kontsevich and Tyler [35], was used for choosing stimuli [36]. This method fits a psychometric function after each newly acquired data point to the whole data set. Simulating the answer of the next trial for each possible acceleration stimulus allows for calculating which stimulus would most change the fit of the psychometric function. This stimulus is considered the most informative and is presented as the next trial. Making use of this method allows for fast and accurate estimation of the psychometric function.

**Fig 3:**
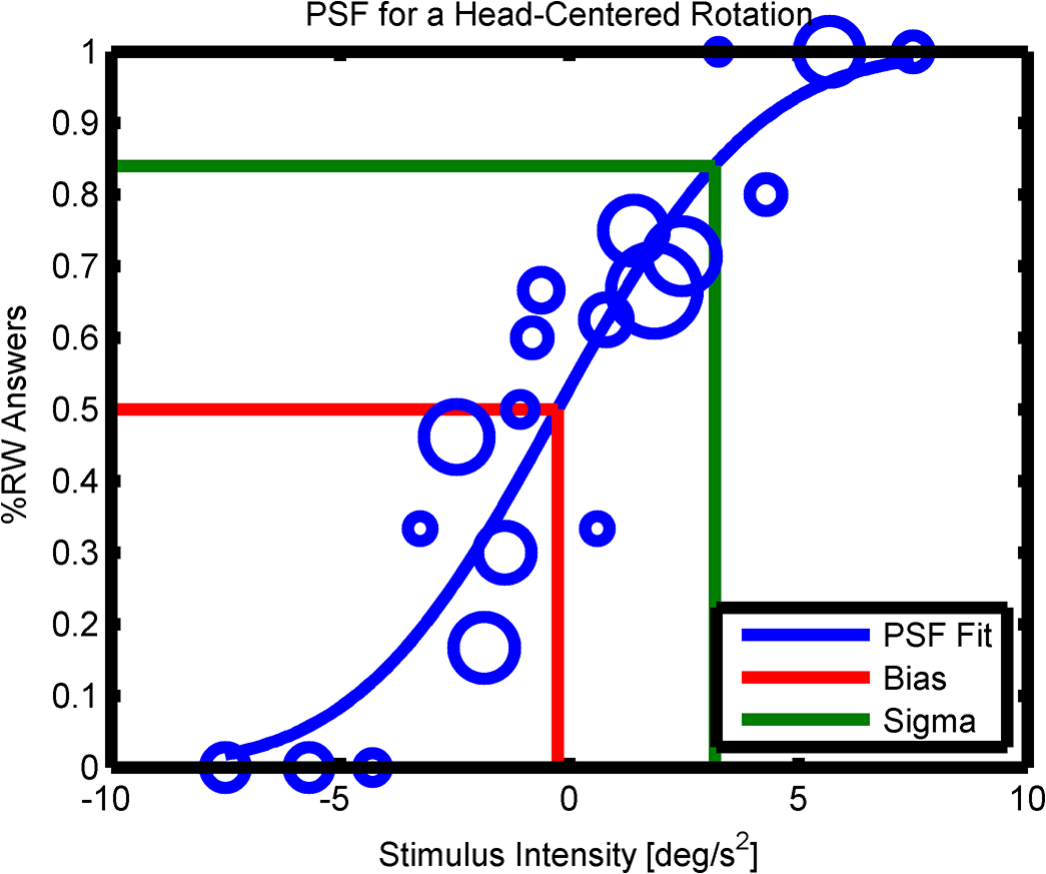
PSF Fit. A psychometric function (PSF) fit: the percentage of rightward responses is plotted against the stimulus intensity (positive intensities correspond to rightward motions). The sizes of the blue circles correspond to how often a certain stimulus was tested. The blue line shows the fit for the psychometric function based on maximum likelihood estimation.

Data for a discrimination task can be analyzed by fitting a psychometric function ranging from 0% rightward answers to 100% rightward answers. This allows for an estimate of the point of subjective equality between leftward and rightward motions (the bias μ) as well as an estimate for the discrimination threshold σ (Fig 3). The psychometric function was modeled as a cumulative normal distribution with the two parameters μ and σ: $F\left(x|\mu ,\sigma \right)=\frac{1}{\sigma \sqrt{2\pi }}\int_{-\infty }^{x}e^{\frac{-{\left(t-\mu \right)}^{2}}{2\sigma^{2}}}dt$. The best fitting parameters given the measurements were found by maximizing the log-likelihood of the model: $LL=\sum_{i=1}^{150}\left[response_{i}\cdot \text{log}\left(F\left(stimulus_{i}|\mu ,\sigma \right)\right)+\left(1-response_{i}\right)\cdot \text{log}\left(1-F\left(stimulus_{i}|\mu ,\sigma \right)\right)\right]$, where the sum runs over all 150 trials, *response*_*i*_ equals 1 if the response was rightward and 0 otherwise, and *stimulus*_*i*_ is the stimulus intensity of the i-th trial.

### Modeling

It is unclear exactly how rotational and tangential accelerations are combined in order to solve the direction discrimination task during eccentric rotations. The discrimination thresholds given only rotational cues (R = 0.0, *σ*_*rot*_) and only translational cues (R = ∞, lateral translation, *σ*_*trans*_) describe the sensitivity of the respective sensor. During eccentric rotations both of these cues (rotational and translational) are present. Our aim is to model the performance during the eccentric rotations (*σ*_*ecc*_) based on the discrimination thresholds *σ*_*trans*_ and *σ*_*rot*_ and the radii of the eccentric rotations. In order to understand how the discrimination thresholds change with increasing radius we propose two plausible models and test which one best fits the data. For the sake of simplicity of notation, thresholds σ within this section are assumed to be given in acceleration units, whereas the results will be reported in velocity units since this is what is usually done in the literature. As explained above one can easily convert between these units since peak acceleration equals peak velocity times Pi.

#### Switching model

The switching model is based on the assumption that the discrimination task is solved by either using only rotational or only translational cues depending on which cue is stronger. Assume a head-centered rotation with threshold stimulus intensity *σ*_*rot*_. A participant should perceive approximately 84% of all trials as being rightward motions (definition of *σ*_*rot*_ being the standard deviation of the underlying normal distribution). Going from head-centered to eccentric rotations with the same rotational acceleration intensity *σ*_*rot*_, the motion will also include the tangential acceleration *t*. As described above, rotational accelerations *α* and tangential accelerations *t* are coupled through the radius $r:\;t=r\cdot \alpha \cdot \frac{\pi }{180}$. Once the radius becomes so large that *t* = *σ*_*trans*_, both cues independently should result in 84% of all trials being judged as rightward motions (using either one of the sensors). For bigger radii less rotational acceleration is needed in order to achieve *t* = *σ*_*trans*_ and the translational cue will dominate the discrimination task. The transition radius can be calculated as: $r_{transition}=\frac{\sigma_{trans}}{\sigma_{rot}}\cdot \frac{180}{\pi }$.

This model implies that up to *r*_*transition*_ the discrimination threshold *σ*_*ecc*_ for eccentric motions would be the same as the threshold for head-centered rotations *σ*_*rot*_. Only after the rotational acceleration *α* results in such a strong tangential acceleration *t* (due to the radius *r*) that *t* > *σ*_*trans*_ will the translational cue contribute more to the discrimination task than the rotational cue. Therefore *σ*_*ecc*_ can be modeled as: $\sigma_{ecc}=\text{min}\;\left(\sigma_{rot},\frac{\sigma_{trans}}{r}\cdot \frac{180}{\pi }\right)$, see Fig 2 (black line).

#### Integration model

An alternative model is one where the discrimination task is solved by taking both, rotational and translation cues into account at the same time. This should lead to a better performance for the intermediate radii compared to only relying on one of the two available cues. For small radii the rotational cue should dominate the discrimination process whereas for large radii the strong tangential acceleration should be easier to detect than the corresponding rotational acceleration. In between we would expect a regime in which the cues are equally strong and the benefit of using both cues in terms of task performance is expected to be largest.

An integration framework providing these properties is the maximum-likelihood integrator scheme which has been successfully applied in many different tasks where sensory information is combined in order to come up with an optimal judgment [37–42]. Assuming independent sources of Gaussian noise underlying the direction discrimination performance of translational and rotational motions, and uniform priors, the combined maximum-likelihood estimate is given by [43]:

$$\sigma_{ecc}^{2}=\frac{\sigma_{rot}^{2}\sigma_{trans,r}^{2}}{\sigma_{rot}^{2}+\sigma_{trans,r}^{2}}$$

where $\sigma_{trans,r}=\frac{\sigma_{trans}}{r}\cdot \frac{180}{\pi }$. The conversion of *σ*_*trans*_ is necessary in order to get comparable quantities in terms of rotational accelerations. *σ*_*trans*,*r*_ expresses how much rotational acceleration is needed to get a tangential acceleration equal to *σ*_*trans*_ and therefore it describes the sensitivity to translational motions at the radius *r* in units of rotational accelerations.

For small and large radii the switching and the integration model produce similar results (if they use the same parameters *σ*_*trans*_ and *σ*_*rot*_), since either rotational or translational cues dominate the discrimination task. However, in between there is a range of radii for which the models clearly differ (Fig 2).

Note that *σ*_*ecc*_ is given in rotational units, meaning that *σ*_*ecc*_ indicates how much rotational acceleration is needed in order to be at the threshold level. Therefore in the extreme cases of *r* = 0 (head-centered rotation) and *r* = *∞* (translation) we expect *σ*_*ecc*_(*r* = 0) = *σ*_*rot*_ and *σ*_*ecc*_(*r* = *∞*) = 0, because at *r* = *∞* an infinitesimally small rotational acceleration would already lead to an infinitely large translation. Strictly speaking *σ*_*ecc*_ is not defined for *r* = 0 and *r* = *∞*. We explain these considerations in order to clarify the asymptotic convergence behavior of *σ*_*ecc*_. In case *r* = 0, *σ*_*trans*,*r*_ will become *∞* and therefore the term $\sigma_{\text{rot}}^{2}$ in the denominator of *σ*_*ecc*_ can be neglected. Now the two $\sigma_{trans,r}^{2}$ terms cancel each other out and $\sigma_{ecc}^{2}=\sigma_{rot}^{2}$. In case *r* = ∞, *σ*_*trans*,*r*_ becomes 0, the $\sigma_{\text{rot}}^{2}$ terms cancel each other and $\sigma_{\text{ecc}}^{2}=0$.

## Results

**Fig 4:**
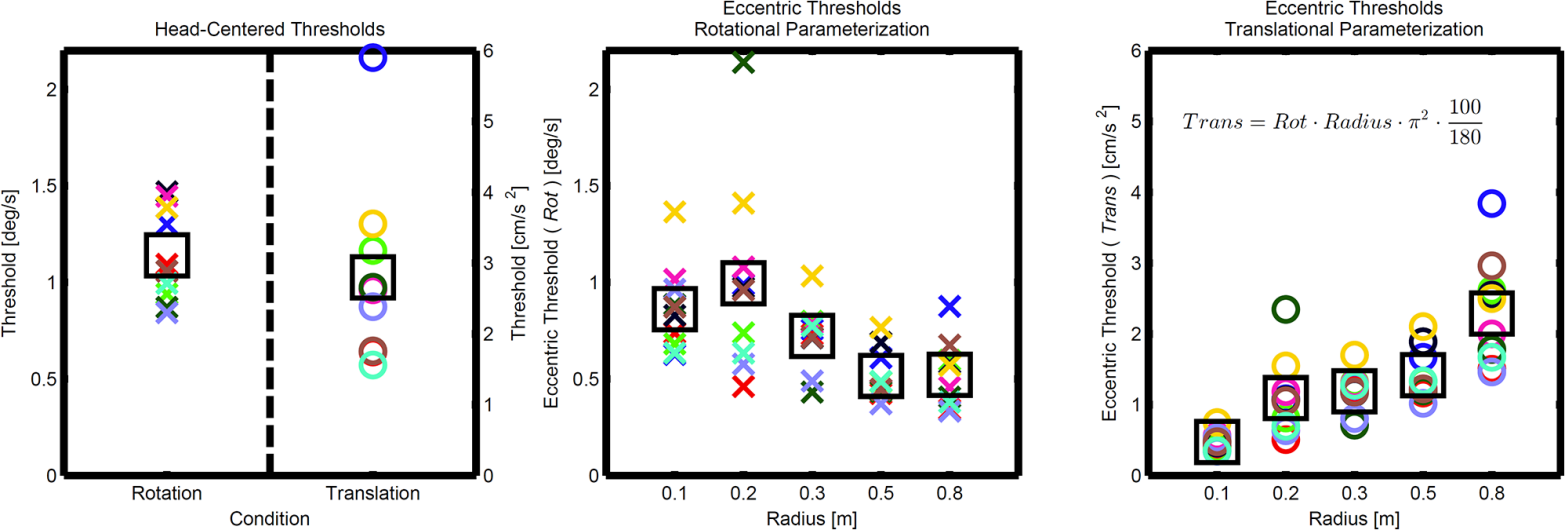
Discrimination Thresholds. The left panel shows the thresholds for the head-centered rotation and translation. Each participant’s data is shown (crosses and circles) together with the arithmetic means (squares). The middle panel shows the thresholds for the eccentric rotation conditions (varying radii) in the rotational parameterization. The right panel shows the same thresholds but in the translational parameterization together with the conversion formula. The effect of the radius on the thresholds can be seen.

The maximum likelihood parameters for the biases and the discrimination thresholds estimated by fitting psychometric functions to each participant’s data for all 7 conditions, as well as the raw data showing every single response can be found in the supplementary material [34]. Table 1 shows the mean biases for all conditions and Fig 4 shows the discrimination thresholds. Note that all means used herein are arithmetic means since our data did not justify the use of geometric means, which are often used for vestibular dominated self-motion perception thresholds (see data distribution in Fig 4). Nevertheless we did the analysis using geometric means as well and the results did not substantially differ. The discrimination thresholds for the eccentric rotations are shown in both possible parameterizations. One can simply convert between the parameterizations using the formula given in Fig 4. We provide both parameterizations to illustrate their relation, but from here on we will use the rotational parameterization only. All statistical analyses were performed using IBM SPSS Statistics 21 with a significance level of p = 0.05.

**Table 1:** Results for the Biases.

Condition	Rotation	r = 0.1	r = 0.2	r = 0.3	r = 0.5	r = 0.8	Translation
	[deg/s]	[deg/s]	[deg/s]	[deg/s]	[deg/s]	[deg/s]	[cm/s^2^]
Mean Bias	0.03	-0.02	-0.04	0.05	-0.01	0.05	0.4
Standard Error	0.13	0.04	0.06	0.05	0.04	0.04	0.2

One-sample t-tests showed no significant deviations of the biases from zero in any of the conditions. A one-way repeated measures ANOVA was conducted to test for an effect of radius on discrimination thresholds in all rotation conditions. There was a statistically significant effect of radius on discrimination thresholds, *F* (5, 5) = 20.47, *p* = .002; Wilk's Λ = 0.047, partial η^2^ = .95.

### Model Predictions

**Fig 5:**
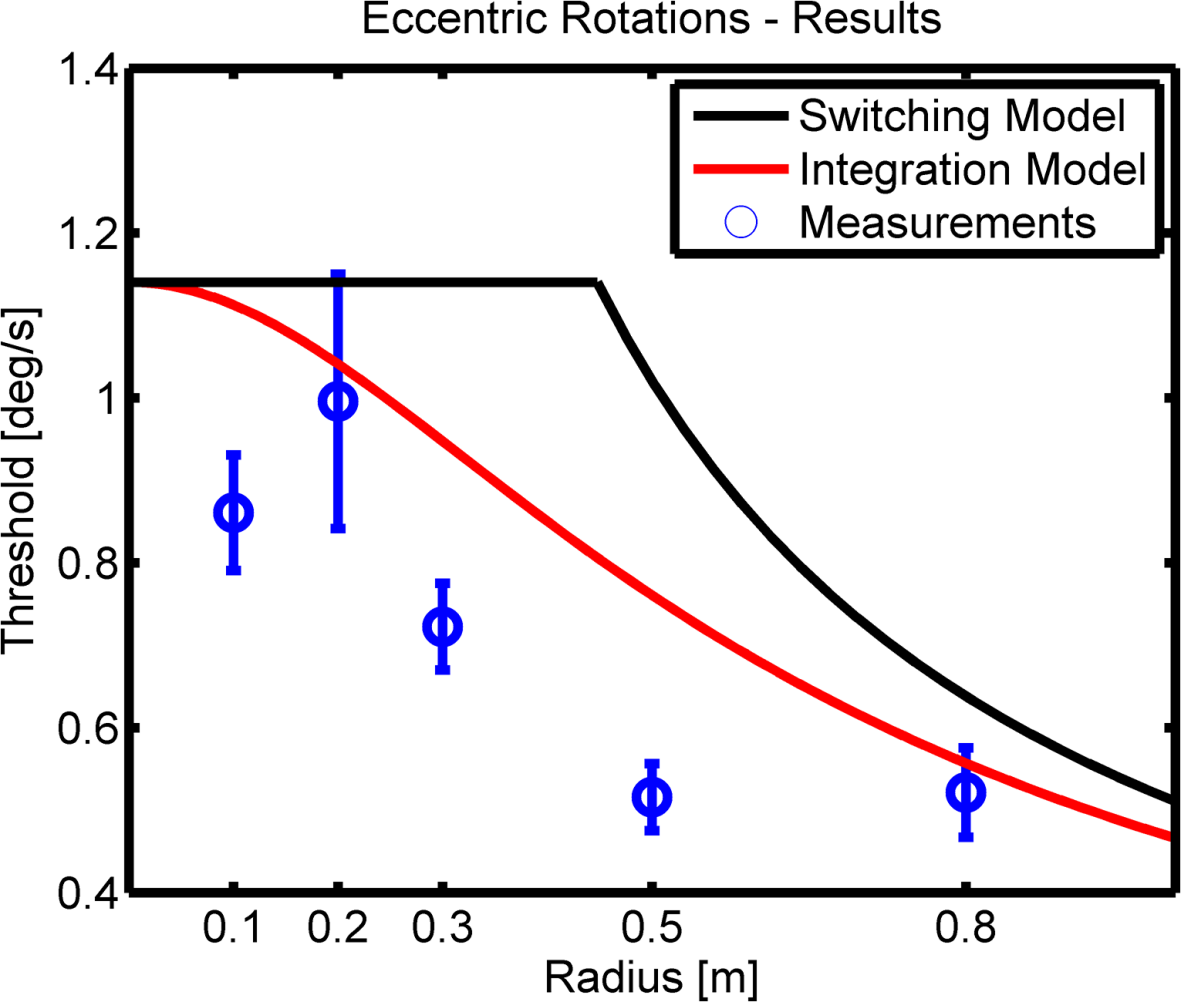
Model Results. Mean results for the eccentric discrimination thresholds together with standard errors are shown (blue). Model fits are shown in black for the switching model and in red for the integration model. It can be clearly seen that the integration model predicts the eccentric thresholds better, but 3 out of 5 thresholds are even significantly lower than what the optimal integration model predicts.

The predictions for the eccentric thresholds based on the integration and the switching model are shown together with the measurements in Fig 5. It can be seen that the integration model (red) better fits all the measurements (blue) than the switching model (black). However, it can also be seen that only 2 out of 5 of the measured thresholds are actually well predicted by the integration model. In order to quantify this, one-sample t-tests were performed testing if the measured threshold distribution at a given radius significantly differs from the integration model prediction for that radius. For *r* = 0.1, 0.3 and 0.5*m* we find significant differences *p* = .006, .002 and *p* < .001. Whereas for *r* = 0.2 and 0.8*m* there are no differences *p* = .78 and .53.

## Discussion

We measured 10 participants' ability to discriminate leftward from rightward motions. Head-centered yaw rotation, lateral translation and 5 eccentric rotations with the rotation center located behind the participant were tested. Psychometric functions (cumulative normal) were fit to the data in order to extract each participant’s bias and discrimination sensitivity. The mean biases did not significantly differ from zero, and, as expected, the discrimination sensitivity increased with increasing radius. This increase is likely due to the fact that eccentric rotations with a given rotational acceleration exhibit tangential accelerations that increase with the radius. These tangential accelerations can be perceived in addition to the rotational accelerations, and due to this increase in sensory information it becomes easier to solve the task.

To our knowledge this is the first study that systematically investigates the changes in thresholds with increasing radii and therefore it represents an important step towards a better understanding of the perception of more complex motions stimulating the SCC and the OO at the same time. Such motions occur in driving or flight simulators for example and the motion cueing algorithms of the simulators make use of perceptual thresholds in order to optimize the perceived motions. Currently tilt thresholds which are based on head-centered measurements are often used for limiting simulator motions [44]. However, often it would be more realistic to use thresholds that are not based on head-centered but eccentric rotations. Our findings could directly be applied in order to understand how thresholds change with eccentricity and therefore might lead to better motion cueing algorithms. Future research should investigate whether these results are generalizable and whether similar improvements in thresholds can also be found for other types of motions that concurrently stimulate both the SCC and the OO.

Our findings show that task performance increases (thresholds decrease) for concurrent SCC and OO stimulation. These findings are especially interesting given that other previous research on concurrent stimulation found performance decreases in motion detection and reproduction tasks [9,16]. Note that those tasks were different in that participants did not make judgments about their whole motion percept, but were asked to judge individual components of the whole motion to which they might not have independent access. Therefore sensory integration seems to be task dependent and further research is necessary to better understand why this is the case.

Our results suggest that the information from the SCC and the OO are integrated during eccentric rotations, resulting in better performance for a direction discrimination task as compared to switching between one of the two sensors. This represents an interesting finding that is in line with results from VOR research [13] and many other examples of integration of sensory information for perceptual tasks. However, most of the previous perceptual research in humans has focused on visual-vestibular integration and did not look at integration within the vestibular system for direction discrimination tasks. Therefore our research adds valuable perceptual data showing how information between the organs of the vestibular system is integrated. The paradigm of eccentric rotations allows for systematic tuning of the ratio between translational and rotational cue intensity and therefore it represents an interesting task which could be helpful in bridging the gap between psychophysical and physiological accounts of cue integration [45].

We showed that an integration model predicts our data better as compared to a switching model. However, some of the measured thresholds were even lower than what an optimal integration model would predict. This hints at an additional source of information that is informative about the direction of motion. Our model assumes that there is independent access to the tangential component of the gravito-inertial force (GIF), but this is likely not the case. The information of the OO about the GIF is combined with information of the SCC in order to achieve the GIF resolution [17]. However at threshold level this might not be easily possible, because the contributing signals from the OO and SCC are close to the noise level. Nevertheless our data suggests that the information from the two sensory organs are combined and task performance increases even beyond what one would expect from combining tangential and rotational acceleration alone. In case GIF resolution would not be possible we would have rather expected the opposite effect: a worsening of task performance, because no independent access to the constituting signals would be possible. As discussed in the introduction there is also a centrifugal component of the stimulus which could generate the feeling of pitch in the participants. Although this potential pitch sensation would not be informative about the direction of motion we still want to quantify its contribution. At threshold level the highest centrifugal acceleration *f* occurs for the *r* = 0.8m radius condition: *f* = *r* ⋅ *ω* ^2^ = 7 ⋅ 10^−5^ *m*/*s* ^2^, where *ω* is the rotational velocity at threshold level in radians per second. This acceleration is so little that it would only produce a tilt of the gravito-inertial vector of ${\text{tan}}^{-1}\;\left(\frac{6\cdot 10^{-5}m/s^{2}}{9.81m/s^{2}}\right)\cdot \frac{180}{\pi }=4\cdot 10^{-4}deg$, which is so small that it should not influence task performance. One possible factor that could explain the performance increase, which is not included in our model, could be that there are somatosensory cues that could help discriminating the direction of motion. As mentioned in the introduction, the discrimination of passive whole-body motions in the dark is dominated by the vestibular system, but nevertheless there are also somatosensory cues present which can be used for direction discrimination and could explain the additional performance increase [4]. These are possibilities, but we do not know for certain why there is more than optimal integration of the cues and more research is needed in order to better understand the behavior of thresholds for eccentric rotations with radii in the range where integration can be observed. In summary we can say that cues from both vestibular organs are integrated which yields significant performance increase that can be observed as compared to what one would expect from a switching model.
